# Prediction of Prostate Cancer Recurrence Using Quantitative Phase Imaging

**DOI:** 10.1038/srep09976

**Published:** 2015-05-15

**Authors:** Shamira Sridharan, Virgilia Macias, Krishnarao Tangella, André Kajdacsy-Balla, Gabriel Popescu

**Affiliations:** 1 Quantitative Light Imaging Laboratory, Department of Bioengineering, Beckman Institute of Advanced Science and Technology, University of Illinois at Urbana-Champaign, 405 N. Matthews Avenue, Urbana, IL 61801, USA; 2 Department of Pathology, University of Illinois at Chicago, 840 S. Wood Street, Chicago, IL 60612, USA; 3 Department of Pathology, Christie Clinic, University of Illinois at Urbana-Champaign, 1400 W. Park Street, Urbana, IL 61801, USA; 4 Quantitative Light Imaging Laboratory, Department of Electrical and Computer Engineering, Beckman Institute of Advanced Science and Technology, University of Illinois at Urbana-Champaign, 405 N. Matthews Avenue, Urbana, IL 61801, USA

## Abstract

The risk of biochemical recurrence of prostate cancer among individuals who undergo radical prostatectomy for treatment is around 25%. Current clinical methods often fail at successfully predicting recurrence among patients at intermediate risk for recurrence. We used a label-free method, spatial light interference microscopy, to perform localized measurements of light scattering in prostatectomy tissue microarrays. We show, for the first time to our knowledge, that anisotropy of light scattering in the stroma immediately adjoining cancerous glands can be used to identify patients at higher risk for recurrence. The data show that lower value of anisotropy corresponds to a higher risk for recurrence, meaning that the stroma adjoining the glands of recurrent patients is more fractionated than in non-recurrent patients. Our method outperformed the widely accepted clinical tool CAPRA-S in the cases we interrogated irrespective of Gleason grade, prostate-specific antigen (PSA) levels and pathological tumor-node-metastasis (pTNM) stage. These results suggest that QPI shows promise in assisting pathologists to improve prediction of prostate cancer recurrence.

The Surveillance, Epidemiology, and End Results (SEER) program by the National Cancer Institute (NCI) estimates that while 233,000 men will be diagnosed with prostate cancer in 2014 in USA alone accounting for 14.0% of all cancer cases, the number of men who will die of the disease is 29,480 accounting for 5.0% of all cancer deaths^1^. Because most prostate cancers are not lethal, active surveillance is a desirable treatment option for patients presenting with localized prostate cancer, low prostate specific antigen (PSA) levels, and low risk according to the D’Amico risk category (see Supplemental Information for a brief review of this metric)^2^. However, radical prostatectomy reduces the risk of bone metastasis and mortality among patients in the intermediate and high risk categories^2^. Studies have shown that the risk of biochemical recurrence, which is defined as increasing serum PSA levels, is around 25% in men who undergo radical prostatectomy, whereas the risk of prostate cancer specific mortality in the same group is 7–12%^3^,^4^,^5^.

Clearly, a method capable of forecasting recurrence is highly desirable. The commonly used tools to predict biochemical prostate cancer recurrence after prostatectomy, Kattan nomogram and Cancer of the Prostate Risk Assessment (CAPRA-S score), have c-index values ranging from 0.76–0.81 (see Supplemental Information for a review of this metric)^6^,^7^. Here we studied pairs of subjects with similar CAPRA-S scores but different outcomes; one member of the pair had biochemical recurrence while the other one did not. In our study, we targeted the groups where existing methods have a discriminatory ability of 0.5. In essence, we used difficult cases where these methods fail to identify patients at high risk of recurrence after prostatectomy. We used quantitative phase imaging (QPI) to measure scattering anisotropy in the stroma adjoining glands in prostatectomy tissue microarrays. We were able to differentiate between recurrent and non-recurrent groups with an area under the receiver-operating characteristic curve (AUC) of 0.72 in 181 difficult cases (3–4 cores per case) regardless of age of the patient, pathologic staging and Gleason grade scores. Our technique exploits *light scattering* signatures in the stroma as a predictive marker.

Light *scattering* rather than *absorption* is the physical phenomenon that renders our bodies opaque to visible radiation. Elastic light scattering, i.e., modification of the direction of propagation without change in wavelength, is induced by the inhomogeneity of tissue at multiple spatial scales. Measuring certain properties of the scattered field (e.g., angular distribution, optical spectrum) informs on the morphology of healthy and diseased tissues. Thus, solving a scattering *inverse problem* holds valuable diagnosis potential. Scattering differences measured using optical coherence tomography have been used as a diagnostic measure for *in-vivo* cancer diagnosis in bladder, esophagus, skin, uterus, stomach and breast^8^,^9^,^10^,^11^,^12^,^13^,^14^. These studies concluded that scattering was stronger in cancerous regions when compared to normal tissue. Studies on colorectal and pancreatic cancer using enhanced backscattering spectroscopy and partial wave spectroscopy have shown the potential of scattering parameters in studying the field effect of cancer^15^,^16^. However, scattering signatures in the stroma and the epithelium cannot be assessed separately using these methods due to low spatial resolution.

While carcinoma itself is uncontrolled proliferation of epithelial cells, it also leads to many changes in the stromal microenvironment with positive feedback into epithelial growth promotion. The importance of stroma has been documented in mouse models, where the implanted cancer is more aggressive when the xenograft has both cancerous epithelium and fibroblasts^17^. The study of stroma as a separate entity is relatively new in optical diagnosis. Second harmonic generation (SHG) studies on ovarian and breast stroma showed that malignant tumors exhibit greater organization in the collagen fibrils with a more uniform orientation^18^,^19^. Further SHG studies in breast showed that the linear orientation of collagen in a direction perpendicular to the cancerous glands, also known as the TACS-3 signature, is associated with poor prognosis in breast cancer^20^. In malignant ovarian tissue, it was shown that, due to this perpendicular alignment, the stromal region adjoining the gland in a parallel orientation show a fractionated appearance^19^. Importantly, light scattering by shorter filaments results in a more isotropic angular distribution. However, structural information obtained using SHG might be incomplete since the signal can only be generated by non-centrosymmetric structures. Additionally, SHG signal amplitude is largely qualitative, as it depends not only on tissue structure, but also the phase matching condition, which cannot be controlled.

Spatial light interference microscope (SLIM) is a QPI technique, central to our approach (see Fig. 1A and Refs. 21,22). SLIM uses a commercial phase contrast microscope and white light illumination, resulting in nanometer scale sensitivity to optical pathlength shifts^23^. In essence, SLIM combines phase contrast microscopy with holography. The instrument was programmed to scan microscope slides containing 320–360 individual cores, as illustrated in Fig. 2. The resulting SLIM image contains rich information about tissue morphology, with the glandular epithelium and stroma structures clearly resolved (Fig. 3). This allows us to interrogate scattering changes specific to prostate stroma. In the past, SLIM has shown potential for prostate cancer diagnosis^21^. A question of great importance in prostate cancer is the prediction of a patient’s cancer recurence. In this paper, we used an unstained prostate tissue microarray containing prostatectomy samples of patients with and without biochemical recurrence of cancer, and studied changes in scattering signatures to identify patients at higher risk for recurrence.

## Results

Analysis of anisotropy in the stroma immediately adjoining the glands from 181 individuals who underwent prostatectomy (89 with recurrence after prostatectomy pair-matched with 89 without recurrence; and 3 additional un-matched recurrent cases) are summarized in Fig. 4. The anisotropy values are displayed as a histogram with a bin width set at 0.01. The anisotropy value in the stromal layer immediately adjoining the glands was lower among patients with recurrence (0.911 ± 0.039) than in patients who did not have recurrence of cancer after prostatectomy (0.935 ± 0.031). This difference in anisotropy values is statistically significant (t-test, p = 7.66 × 10^−6^). The accuracy of anisotropy measurement in the stroma was characterized using the Henyey-Greenstein fit, as shown in Fig. 4C-D. The accuracy estimation is described in more detail in materials and methods.

Anisotropy (g) can be used to distinguish between recurrent and non-recurrent cases with an AUC of 0.72 as shown in Fig. 5. When the prediction threshold was set at g = 0.938, as determined from the ROC curve, recurrence could be predicted with a sensitivity of 77% and specificity of 62%. The threshold value, g = 0.938, is not between the mean values of the two distribution due to the skewness in the g- distribution among the recurrent and non-recurrent groups. The threshold is in the region between the median value of the recurrent (0.921) and non-recurrent (0.945) groups. The CAPRA-S score (see Supplemental Information), which is commonly used as recurrence prediction tool in clinical practice, was calculated for 161 patients (83 recurrent, 78 non-recurrent) from the same set. Twenty subjects were not included in CAPRA-S analysis due to missing values for pre-surgical PSA level, extra-capsular extensions, and/or lymph node status. In those 161 patients, CAPRA-S distinguished between the two groups with an AUC of 0.54. This low performance of CAPRA-S is not surprising because some of the parameters used for CAPRA-S calculation, namely, Gleason score and lymph node status were pair-matched by design.

Pre-surgical prostate specific antigen (PSA) level is one of the parameters used in CAPRA-S calculation and ranges from 0–3 out of the total possible CAPRA-S score of 0–12. This means the PSA level can have a significant impact on a patient’s CAPRA-S score. The cases in our tissue microarray (TMA) cohort were not matched based on PSA levels and PSA showed poor correlation with anisotropy (Pearson r = −0.12). In order to study the effect of PSA on the predictive ability of CAPRA-S score and anisotropy, we compared the performance of the two tools at the PSA ranges used in the CAPRA-S calculations as shown in Fig. 6.

At the PSA range of 0–6 ng/ml, the CAPRA-S parameter failed (AUC 0.4) on the 57 cases (31 recurrent, 26 non-recurrent), whereas anisotropy showed better performance (AUC 0.7). In the intermediate PSA range of 6.01–10 ng/ml and 10.01–20 ng/ml, both CAPRA-S and anisotropy were able to discriminate between recurrent and non-recurrent cases, but anisotropy showed significantly better performance. Anisotropy showed best performance in the PSA range of 10.01–20 ng/ml with an AUC of 0.88 in 36 cases (23 recurrent, 13 non-recurrent).

In the 18 cases where PSA levels were greater than 20 ng/ml, both CAPRA-S and anisotropy showed poor performance. Our data shows a decrease in anisotropy value in the stroma adjoining glands among all individuals with pre-surgical PSA levels greater than 20 ng/ml, irrespective of recurrence status. This decrease in anisotropy among non-recurrent individuals contributes to the low discrimination of our method. We believe that high PSA levels influence the morphology of stroma such that it causes increasing fractionation and non-uniform swelling of stromal fibers (illustrated in Fig. S6 in the supplemental information). Our observations are consistent with previous studies that have implicated PSA in regulating prostate stromal cell growth by modulating interactions with growth factors and cytokines^24^,^25^. The reason CAPRA-S failed on cases with high PSA levels is that levels of PSA > 20 ng/ml corresponds to 3 CAPRA-S points out of the maximum 12. Hence it over-estimates the CAPRA-S score among individuals without recurrence but high pre-surgical PSA levels. Note that, CAPRA-S is expected to fail among non-recurrent patients with high PSA levels, as the patient pairs are matched in all other relevant parameters. In all PSA ranges, anisotropy performed better than CAPRA-S and a higher value of anisotropy corresponded to a lower probability of biochemical recurrence of prostate cancer.

## Discussion

It is rather interesting that recurrence is associated with lower g-values. In other words, more serious clinical states are correlated with tissue scattering that appears more isotropic. These findings suggest that the stroma around the recurrent glands is fragmented to smaller subunits than in their non-recurrent counterpart or that there is a loss of collagen fiber alignment in cases of worse outcome. Normal prostate stroma is composed of collagen fibers, smooth muscle cells and fibroblasts, unlike breast and ovarian stroma, which are not smooth muscle-rich^26^. This might also explain why collagen orientation results are markedly different in breast, ovarian and prostate cancers.

We believe that the changes in anisotropy of scattering detected by QPI are consistent with current understanding of prostate cancer biology. In order for cancer to metastasize, malignant epithelial cells breach the basement membrane and invade the stroma. This appears to explain why the changes we detected in stroma are directly adjoining the glands. Stromal invasion also triggers a wound healing process during which various growth factors are secreted and fibroblasts switch to myofibroblastic phenotype for wound closure^27^,^28^,^29^. The inability to successfully close the wound, which was associated with decreased α-smooth muscle actin and desmin expression has been correlated with shorter recurrence time^28^. Fibroblasts and myofibroblasts also continue secreting extra-cellular matrix components (ECM) and proteases for degradation of existing ECM. High levels of stromal protein cleavage factors matrix metalloproteinase (MMP) 2,9 and lower levels of tissue inhibitors of metalloproteinase (TIMP) 1,2 were associated with higher Gleason scores (8–10), higher probability of metastases and lower probability of cure^30^,^31^. Molecular studies have shown the importance of the stroma in cancer aggressiveness. However, studying all molecular expression changes together would involve multiple rounds of immunohistochemistry and other tests which are difficult to quantify or reproduce. On the other hand, QPI characterizes changes in the light scattering caused by morphological changes in the stroma, which are the final result of all the molecular changes associated with cancer progression. The increasingly fragmented appearance of stroma and the increased disorganization in cases with poor prognosis is measured as a decrease in optical anisotropy. Anisotropy shows significant promise in forecasting the recurrence of prostate cancer in individuals who undergo prostatectomy. By combining the label-free nature and nanoscale sensitivity of SLIM, we measure changes in optical scattering that are not measurable using conventional pathology techniques. The samples used in this paper represent cases in which the current widely accepted prognostic tool CAPRA-S fails at predicting the recurrence of prostate cancer. Further studies will determine whether anisotropy can be used to identify high-risk patients in other cohorts and from prostate biopsies, and thus distinguish patients who might benefit from active surveillance instead of prostatectomy.

## Method

### Prostate tissue specimens

We used the National Cancer Institute Cooperative Prostate Cancer Tissue Resource (NCI-CPCTR) tissue microarray (TMA5), which provides both tissue and clinical data associated with patients who underwent radical prostatectomy for treatment of prostate cancer. The tissue was collected at four academic institutions: George Washington University, New York University, University of Pittsburg, and Medical College of Wisconsin. Procedures, policies and protocols for TMA construction and slide preparation are available at the CPCTR website^32^,^33^. The studies have been performed in the United States in accordance with the procedure approved by the Institutional Review Board at University of Illinois at Urbana-Champaign (IRB Protocol Number: 13900).

### TMA cohort

The outcomes TMA set (TMA5) includes pathologic material from 200 paired recurrence and non-recurrence prostate cancer cases (400 altogether). Measurement of prostate-specific antigen (PSA) levels in serum is the most commonly used diagnostic and tumor recurrence marker in prostate cancer. Increasing serum PSA concentrations is considered evidence of clinical recurrence after prostatectomy or radiation treatment. The CPCTR created a Perl-based algorithm to calculate post-treatment PSA outcomes results based on the initial PSA and multiple PSA values obtained after treatment. For the script, PSA recurrence was defined as a single PSA value of greater than 0.4 ng/ml, or a PSA value greater than 0.2 ng/ml with additional subsequent increasing values. Details of the algorithm can be found in this reference 34.

Each patient with biochemical recurrence of prostate cancer was matched by race, Gleason sum score (primary and secondary Gleason grades were also accounted), pTNM stage, and age at radical prostatectomy with a patient who did not show recurrence. All cases had no known metastasis, ≥5 years follow-up, a PSA nadir and at least 5 PSA tests after surgery. Cases were arrayed over five blocks with a single focus of tumor from each patient represented in quadruplicate 0.6 mm cores. Case-control pairs were allocated in the same block. The complete set of five blocks has 1,600 prostate tissue cores. For this study, two 4 μm thick TMA tissue sections were cut from each block at the University of Illinois at Chicago. The thickness was set at 4 μm in accordance with standard practice in pathology. One set was stained with Hematoxylin and Eosin (H&E) for pathology verification and also as a control. An adjacent set was submitted unstained to the Quantitative Light Imaging Laboratory, Urbana, Illinois, for SLIM imaging. This set of slides was subsequently de-paraffinized, cover slipped and imaged using the spatial light interference microscope (SLIM).

### Spatial Light Interference Microscopy (SLIM)

**Figure 1:**
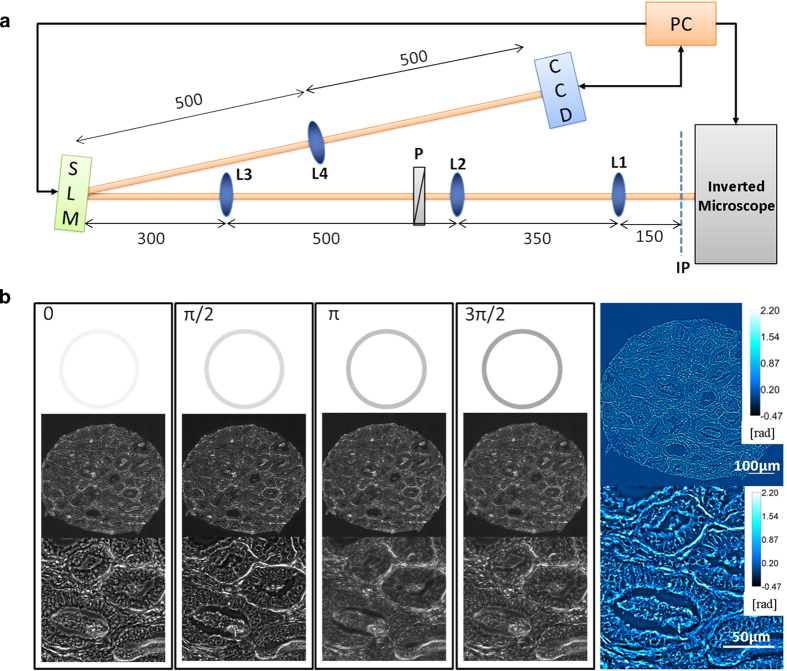
The imaging system. (**A**) The SLIM set-up is an add-on module to a commercial phase contrast microscope. The first set of lenses (L1 and L2) magnify the image to maintain the resolution of the microscope. The Fourier transform of the image plane is projected by lens L3 onto the spatial light modulator (SLM) where the phase pattern is shifted in phase 4 times, in increments of π/2. The lens L4 Fourier transforms the pattern on the SLM and the final image is recorded onto the CCD and stored on the computer. (**B**) Four phase shifted images recorded using a 40X/0.75NA objective with the final quantitative phase image shown on the right. Color bars indicate phase values in radians.

SLIM is developed as an add-on module to an existing phase contrast microscope, as shown in Fig. 1A. The image field outputted by the microscope is Fourier transformed by the lens system L1, L2, L3 onto the surface of a spatial light modulator (SLM). The SLM shifts the phase of the unscattered light with respect to the scattered light, sequentially, in increments of π/2 (see Ref. 21). Lens L4 recreates the image of the sample at the CCD, which records an image for each phase modulation. The four *intensity* images are combined to uniquely render the quantitative *phase* map of the image field, as shown in Fig. 1B. The transverse resolution of SLIM images is 0.4 μm, limited by the numerical aperture of the objective and the spatial path length sensitivity is 0.3 nm.

**Figure 2:**
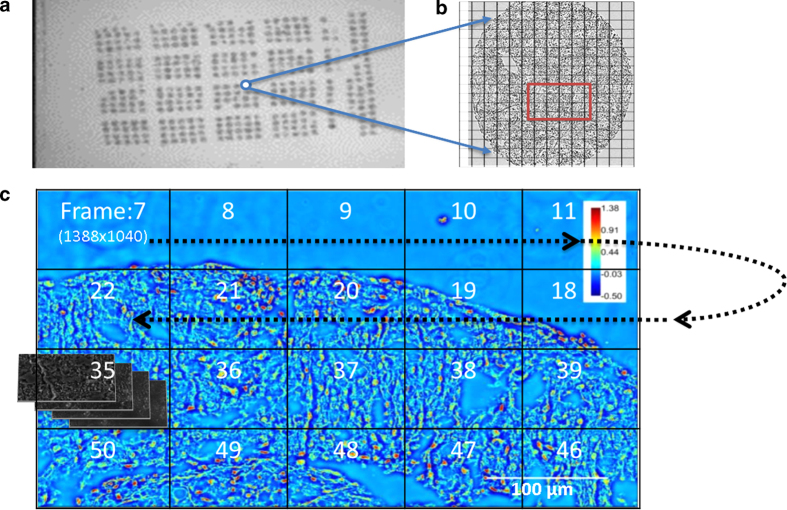
Mosaic SLIM imaging of an unstained tissue microarray. (**A**) Unstained tissue microarray slide. (**B**) The mosaic is set up around the core of interest. (**C**) The recording at each tile proceeds as shown by the arrow. For each 1388 × 1040 pixel SLIM tile, four intensity images are recorded. The phase images are then stitched together using an ImageJ plugin built in our lab.

**Figure 3:**
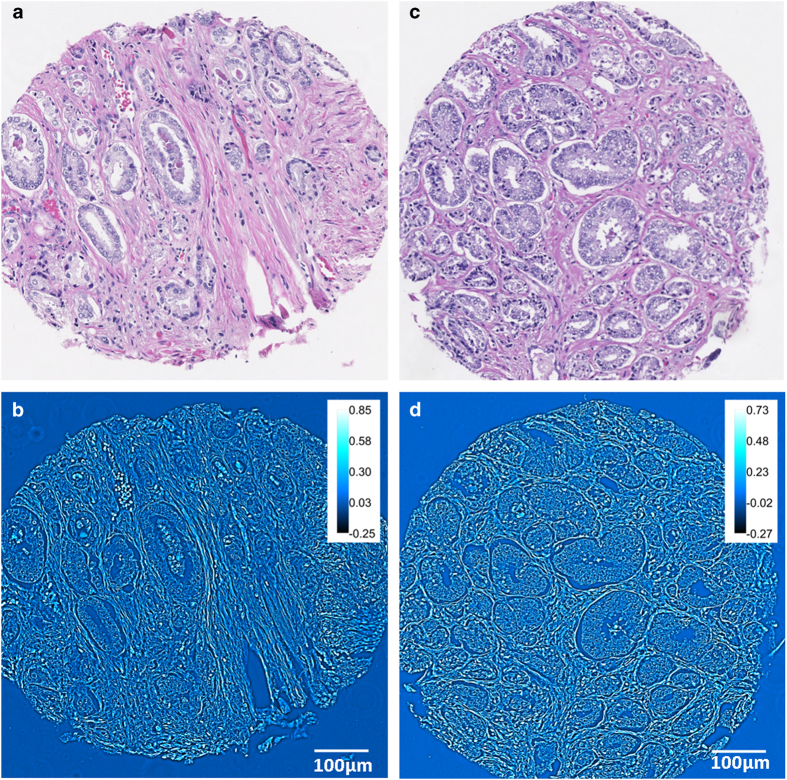
Comparison of H&E and SLIM images. (**A**,**B**) H&E and SLIM images corresponding to a patient who had biochemical recurrence of prostate cancer after undergoing radical prostatectomy. (**C**,**D**) H&E and SLIM images corresponding to the matched twin who did not have cancer recurrence. Both patients had Gleason score 7 (3 + 4) prostate cancer of pT2b stage without seminal vesicle invasion, no extra-prostatic extensions and surgical margins were free of cancer. The H&E images themselves do not provide any information about recurrence.

In order to image pathology slides, the SLIM imaging system was modified to mosaic together large fields of view necessary for diagnosis. The SLM switching, image acquisition, and stage scanning was synchronized via the computer and software developed in house. Before scanning, individual focus points were set at each frame of the mosaic to ensure the entire tissue is in focus. Post-processing code was written in MATLAB and ImageJ to stitch all the tiles of the mosaic together in order to obtain the final quantitative phase image of the tissue. The TMA slides were imaged via a 40X/0.75NA objective as illustrated in Fig. 2. The size of single field of view in the 40X SLIM system is approximately 99 × 74 μm, corresponding to 1388 × 1040 pixels on the camera. The diameter of each tissue core was approximately 0.6 mm, so each core was imaged as a 10 × 10 mosaic which was cropped to 10,000 × 10,000 pixels for analysis. An example of the side-by-side comparison between SLIM images and H&E stained images is shown in Fig. 3. Note that the morphological details contained in the H&E image, e.g., glandular structure, cell nuclei and stromal fiber alignment are recovered in the SLIM image.

**Figure 4:**
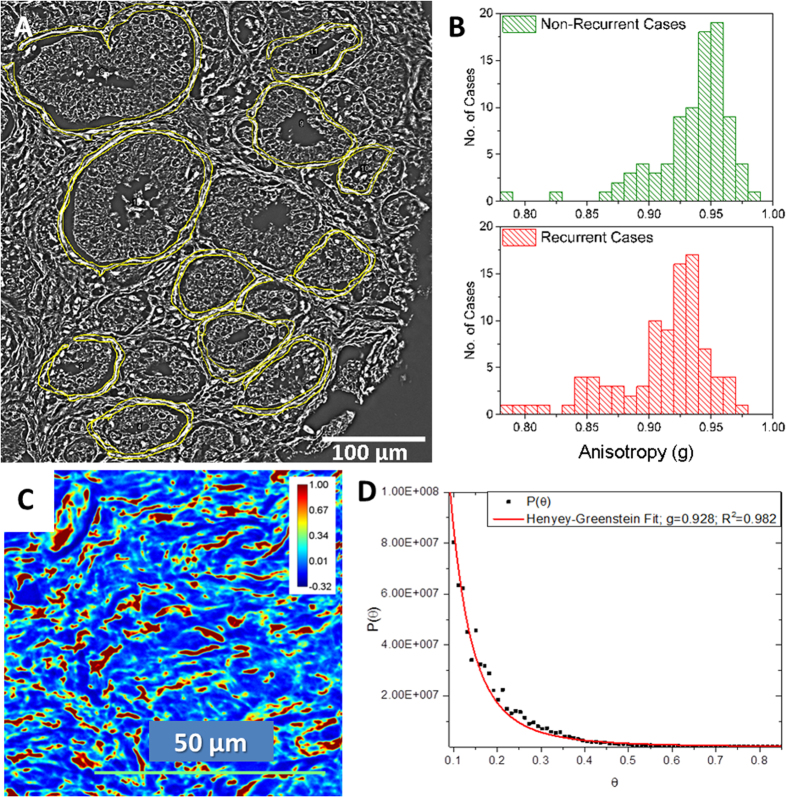
Optical Anisotropy Calculation. (**A**) Optical anisotropy (g) was calculated in the single layer of stroma immediately adjoining multiple glands in each core. (**B**) The histograms show the distribution of anisotropy values among the 89 non-recurrent and 92 recurrent cases. The bin-size on the histogram was set at 0.01. (**C**) SLIM image of a stromal tissue region in the prostate imaged using the 40X/0.75NA objective. Optical anisotropy value calculated using the scattering phase theorem in this tissue region was g = 0.932. (**D**) Anisotropy calculation using Henyey-Greenstein phase function fit of the scattering angular distribution yields g = 0.928.

**Figure 5:**
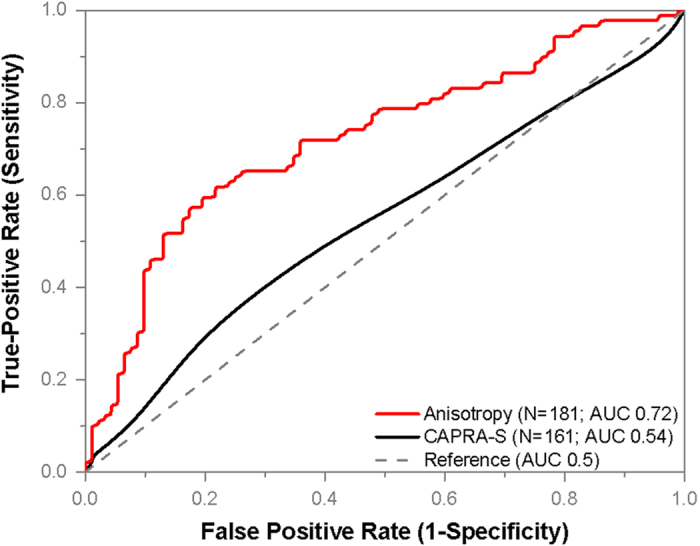
Results for Prostate Cancer Recurrence using Anisotropy of Scattering. Single layers of stroma immediately adjoining 12–16 glands were isolated in SLIM images from each of the 92 recurrent and 89 non-recurrent patients who underwent prostatectomy. The patients in the two groups were matched based on age at prostatectomy, Gleason grade and clinical stage. The optical anisotropy parameter was calculated for each region, as described in Materials and Methods. This parameter separates cases of recurrence from non-recurrent twins with an AUC of 0.72, as shown. Lower values of this index correspond to a greater probability of biochemical recurrence. By using a cut-off value of g = 0.938, we can predict recurrence with a sensitivity of 77% and specificity of 62%. CAPRA-S scores corresponding to 161 patients, 83 recurrent and 78 non-recurrent, showed poor discrimination (AUC 0.54). Twenty cases were excluded in CAPRA-S analysis due to one or more missing parameters for CAPRA-S calculation.

**Figure 6:**
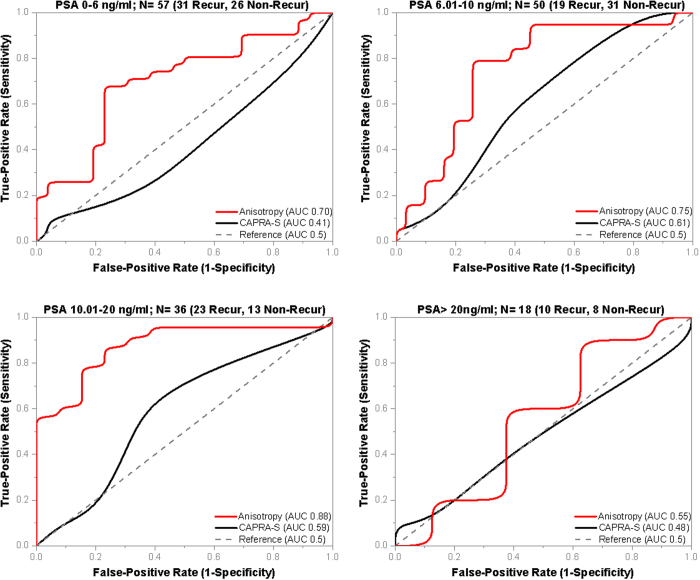
Influence of PSA on Anisotropy Values. Optical anisotropy (g), has poor correlation with pre-surgical PSA levels which prompted the prostate cancer diagnosis (Pearson r = −0.12). The performance of anisotropy and CAPRA-S is compared across various PSA ranges. (**A**) At PSA 0–6 ng/ml, anisotropy (AUC 0.7) outperforms CAPRA-S (AUC 0.41), which failed on the 57 cases. (**B**) At PSA 6.01–10 ng/ml, CAPRA-S has the best results (AUC 0.61), but anisotropy (AUC 0.75) still shows better discrimination. (**C**) At PSA 10.01–20 ng/ml, anisotropy (0.88) has the best performance among all the PSA ranges and performs better than CAPRA-S (0.59). (**D**) At PSA > 20 ng/ml, both anisotropy (AUC 0.55) and CAPRA-S (AUC 0.48) show poor discrimination.

Stromal regions directly adjoining randomly selected 12–16 malignant glands per patient were selected for analysis. A Wacom tablet and the region of interest (ROI) feature on ImageJ were used to manually select the stromal ROIs as shown in Fig. 4A. The size of the ROI is subject to variation based on the size of the glands. Additionally, the width of stromal fibers can vary due to swelling. Individual stromal fibers and separation between adjacent fibers are clearly visible in SLIM images enabling accurate segmentation of the stromal fiber adjacent to the gland. For each stromal region, we calculated the *optical anisotropy, g*, which is a measure of directionality of light scattered by tissue, as detailed below.

### Optical Anisotropy Calculation

The *scattering-phase theorem*^35^ *states that* the anisotropy factor can be computed from quantitative phase images as


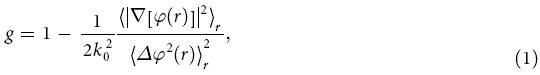


where k_0_ = 2π/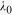 is the wavenumber, 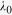 = 552 nm is the center wavelength of white light used in our imaging, r is the stromal region over which optical anisotropy is calculated, 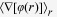 is the mean phase gradient intensity, and 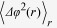 is the phase variance. Eq. 1 is derived using the regular thin object approximation for QPI, which gives the expected phase shift proportional with thickness. However, g is a property of the tissue bulk, not of the particular slice. Therefore, as long as the out-of-focus light is negligible, i.e., the phase measurement integrates over the entire tissue thickness, g is independent of thickness.

Once the quantitative phase image of the core was available, a map of the gradient was computed using ImageJ. In our study, we calculated g in the stromal layer immediately adjoining the glands, as illustrated in Fig. 4A. This stromal region was the region of interest, ‘r’, in equation (1). The mean phase gradient intensity and phase variance were computed in the stromal region of interest and finally the value of g was obtained.

We calculated the accuracy in the measurement of anisotropy retrieved from the scattering phase theorem by comparing it with g-values obtained from fitting the scattering angular distribution with the Henyey-Greenstein phase function. The Henyey-Greenstein distribution is^36^:


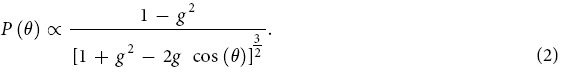


The g-value obtained with the Henyey-Greenstein fit over the tissue region shown in Fig. 4C is g = 0.928, with fit accuracy R^2^ = 0.982 (Fig. 4D). The g-value over the same region calculated using the scattering phase theorem, is g = 0.932.

For the purpose of prognosis and diagnosis, *precision* in the measurement of g is more critical than *accuracy*. In order to estimate the errors in our g calculations, we considered an area of background, with no tissue, and quantified the effect of the background phase noise upon the resulting g-values. Let us consider


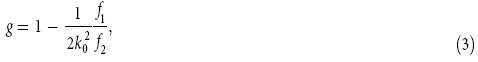


where 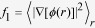 is the average phase gradient intensity squared over a given tissue region and 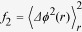 is the phase variance squared over the tissue region.

The error in our measurement is given by


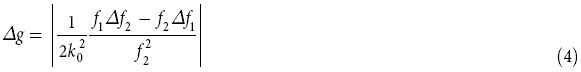


where 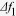 and 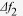
are the standard deviations due to phase noise in 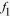 and 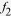, respectively. In order to evaluate 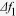 and 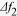, we selected a region of the SLIM image with no tissue, i.e., background, and computed the standard deviations of 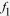 and 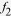 due to noise. As a result, we obtained 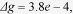 which describes the error in our measurements due to spatial phase noise. This low value is the direct result of the absence of speckles in SLIM imaging due to the white light illumination.

## Author Contributions

S.S. performed the experiment and analyzed experimental data. V.M., A.K.B. and K.T. provided pathology expertise. V.M. and A.K.B. provided materials and reagents. S.S., V.M., A.K.B. and G.P. wrote the paper.

## Additional Information

**How to cite this article**: Sridharan, S. *et al*. Prediction of Prostate Cancer Recurrence using Quantitative Phase Imaging. *Sci. Rep.* **5**, 9976; doi: 10.1038/srep09976 (2015).

## Supplementary Material

Supplementary Information
