# Supplementary material for: Prediction of Prostate Cancer Recurrence Using Quantitative Phase Imaging
Source: Sci Rep. 2015 May 15;5:9976. doi: 10.1038/srep09976 (PMC4432311; doi:10.1038/srep09976)
Supplement: Supplementary Information [file srep09976-s1.pdf]

Supplemental Information

Prediction of Prostate Cancer Recurrence using Quantitative Phase Imaging

Shamira Sridharan[1], Virgilia Macias[2], Krishnarao Tangella[3], André Kajdacsy-Balla[2] and Gabriel Popescu[4,*]

1. Gleason Grading

The Gleason grade is the most widely used grading scheme in prostate cancer. The system was developed by Dr. Donald Gleason in the 1960s and is based on glandular differentiation seen in hematoxylin and eosin (H&E) stained slides [1, 2]. Pathologists determine Gleason grade based on glandular presence and differentiation in stroma [1, 2]. The Gleason score has been proven to be an indicator of tumor size, metastasis, treatment and outcome [2-5].

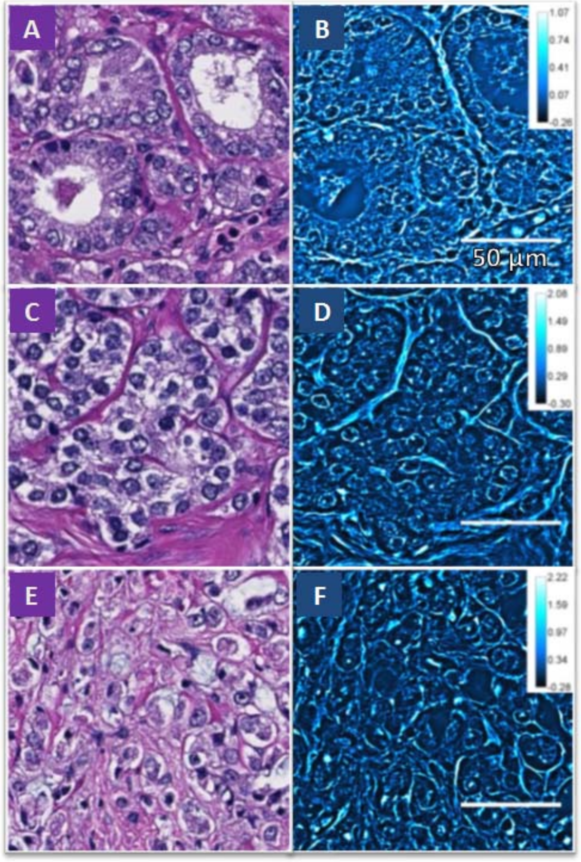

Figure S1: *A side-by-side comparison of H&E and SLIM images of the same tissue microarray cores show that SLIM images reveal a lot more information about the tissue due to the intrinsic contrast in tissue. Single strands of stroma stand out in SLIM images as opposed to the uniform pink distribution seen in H&E images. In the zoomed in version of the image seen in figures* ***A & B****, the stroma separating glands pops out clearly in SLIM images enabling the classification of the glands as grade 3. Side-by-side comparison of Gleason grade 4 tissue microarray cores shown in* ***C & D*** *once again shows the ability of SLIM to detect stroma allowing for better visualization of fusing glands and stromal invasion allowing for accurate Gleason grading. Also, smaller structures within nuclei are more prominent. In the Gleason grade 5 images shown in* ***E & F****, the loss of glandularity and presence of epithelial cells in stroma are clearly visible in the SLIM images*

The variation in grades on a scale from 1-5, in order of increasing severity, is based on glandular differentiation and glandular presence in stroma [1, 2, 6, 7]. The primary grade is the pattern present in maximum biopsy area and the secondary grade is the second most prominent pattern. The two grades are added to provide a Gleason score of 1-10.

In current clinical practice, grades 1 and 2 are rarely diagnosed. Gleason grade 3 glands are medium to small size, singular with infiltrating edges as seen in Figs S1A,B which shows a side-by-side comparison of H&E and SLIM images. In a zoomed-in version of Gleason grade 3 in figure S2A, stroma between adjacent glands is clearly visible, thus, aiding the determination of the grade [2]. Gleason grade 4 consists of small glands that are fusing into one another (Figs S1C, D; Fig S2B) [2]. Gleason grade 5 represents the most severe cancer where glandular architecture is lost and epithelial cells are distributed individually or in sheet like patterns in the stroma (Figs S1E, F) [1]. In the zoomed-in version in Fig S2C, no glands are visible and epithelial cells can be seen between stromal fibers.

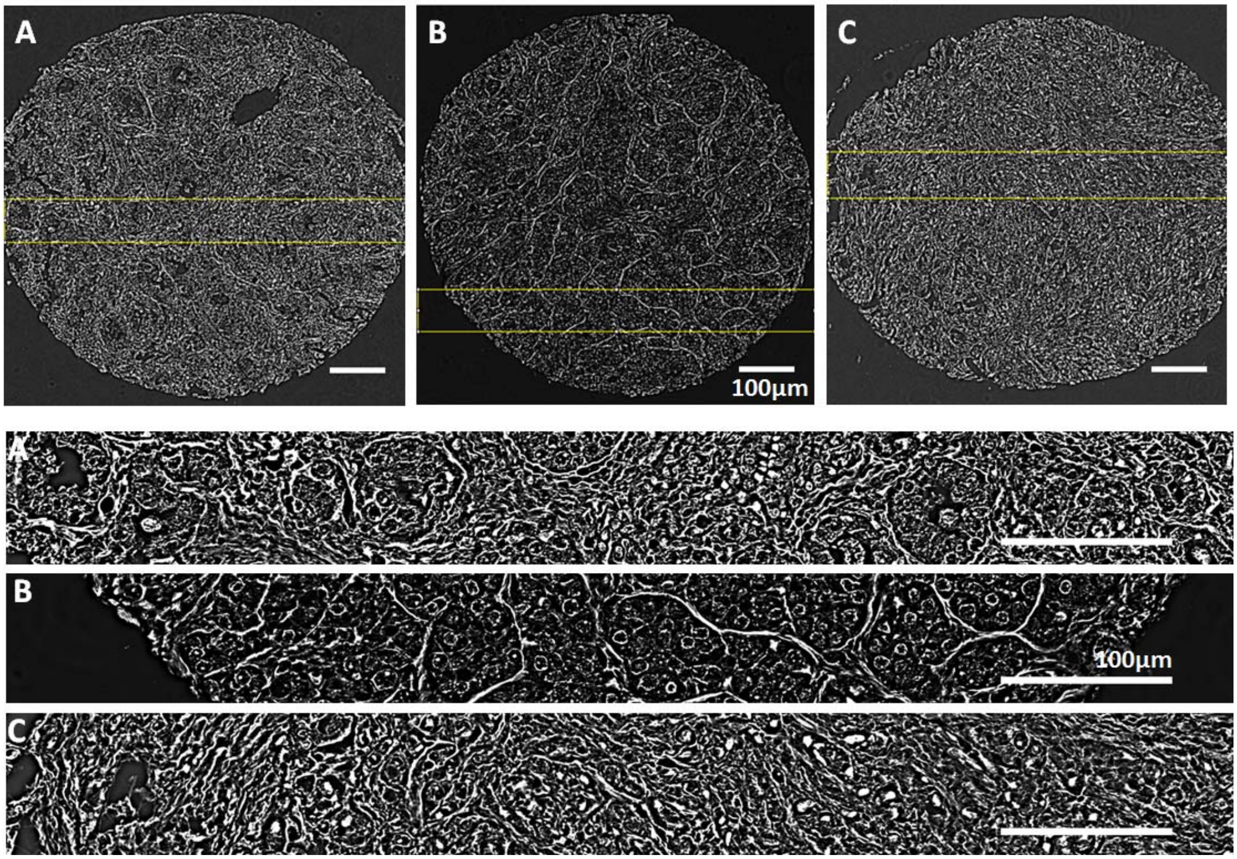

Figure S2: Zoomed in SLIM images of various Gleason grades. *Fig A: Gleason grade 3 pattern with medium sized individual glands separated by stroma. Fig B: Gleason grade 4 pattern with small merging glands showing loss of stromal separation. Fig C: Gleason grade 5 pattern with no visible glands and individual cells interspersed in the stroma.*

2. D'Amico Risk Classification

The D'Amico risk classification is based on the combination of clinical parameters such as prostate specific antigen (PSA) levels measured in blood, Gleason score on the biopsy and prostate tumor size (T) as measured in either a digital rectal exam or trans-rectal ultrasound. There are three categories [8]:

1. D’Amico low risk category: Blood PSA level ≤ 10 ng/ml, Gleason score ≤ 6, T1-T2a. (T1: Tumor was an incidental finding that is not palpable; T2a: Tumor is in less than one half of one side of the prostate)
2. D’Amico intermediate risk classification: Blood PSA level of 10-20 ng/ml, Gleason score 7, T2b. (T2b: Tumor is confined to one side of the prostate, but more than one half of one lobe)
3. D’Amico high risk classification: Blood PSA level > 20 ng/ml, Gleason score ≥ 8, T2c-T3a. (T2c: Tumor is in both sides of the prostate; T3a: Tumor shows extra-capsular extension)

As displayed in the profile distribution histograms in Figure S3, the majority of the cases used in our study are, by design, from the D’Amico intermediate risk category.

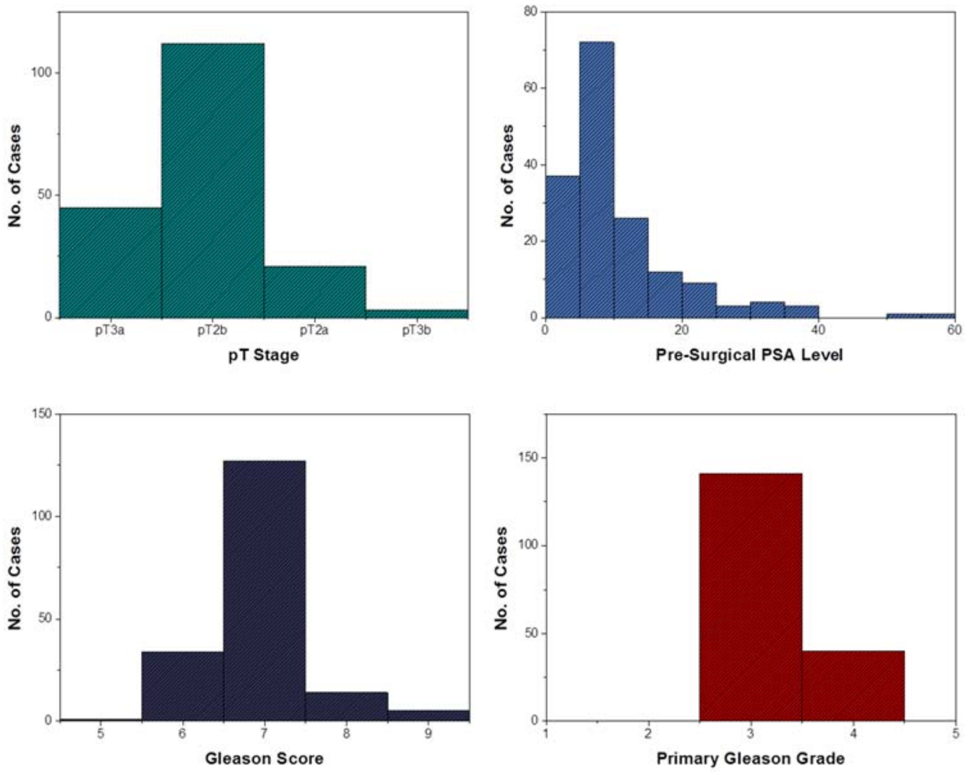

Figure S3: Patient Demographics. *The histograms show the demographics associated with 181 prostatectomy cases used for our study based on various parameters such as the pT stage, pre-surgical PSA levels, Gleason score and primary Gleason grade.*

3. CAPRA-S Score

The Cancer of the Prostate Risk Assessment (CAPRA-S) score is a commonly used post-radical prostatectomy prostate cancer recurrence risk assessment tool, which is described in detail elsewhere [9]. It assigns differential weightage to PSA levels before surgery, pathological Gleason score, extra-capsular extension, surgical margin status, seminal vesicle invasion and lymph node invasion. The weightages are added up to provide a score that determines the risk of biochemical recurrence of prostate cancer.

4. Receiver Operating Characteristic (ROC)

The ROC curve plots the sensitivity (true positive rate) against 1-specificity (false positive rate). In order to plot an ROC curve using binary data, incremental cut-off or threshold values are set at which the true

positive and false positive rates are determined and plotted. The area under the ROC curve (AUC) (this is equivalent to c-index in a binary outcome, however, c-index is calculated differently) represents the accuracy of the classification. An AUC of 1 corresponds to a perfect classification method whereas an AUC of 0.5 corresponds to random guess, like a coin toss.

5. Anisotropy factor, g

The anisotropy factor is the average cosine of the scattering angle, $g = \langle \cos(\theta) \rangle$.

Using the *scattering-phase theorem,* we calculated g across a specimen in terms of the gradient and variance of the spatial phase distribution (Eq. 1 in text). The anisotropy values are averaged across the stroma adjoining multiple glands from 3-4 cores per patient to obtain the final g-value for each patient.

Figure S4 illustrates the low- and high-values of g: more isotropic scattering corresponds to lower values of g. Our results indicate that bad outcomes are correlated to lower g-values.

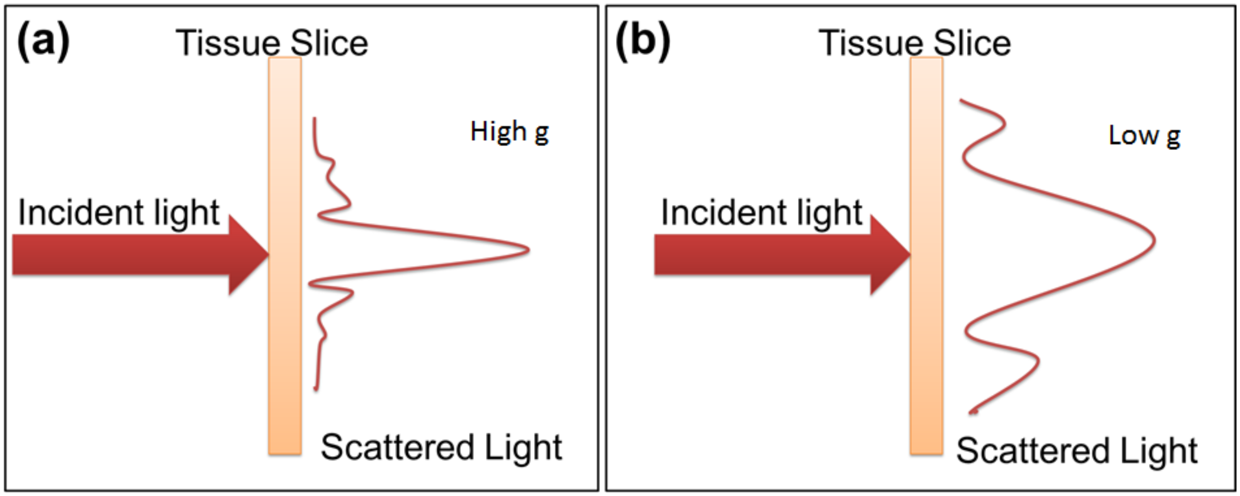

Figure S4: Optical Anisotropy. *Optical anisotropy (g) is defined as the average cosine of the scattering angle in a single scattering event. Optical anisotropy is directly proportional to the square of the magnitude of phase gradient averaged over a tissue region and inversely proportional to the phase variance in the same region. (a) A tissue slice with high anisotropy is primarily forward scattering. (b) Tissue slide with low anisotropy scatters uniformly in all directions.*

The morphology of stroma adjoining glands among patients with high PSA levels (PSA>20ng/ml), is fractionated and shows non-uniform swelling. This causes anisotropy to fail at identifying non-recurrent individuals with high pre-surgical PSA levels as illustrated in Figure S5.

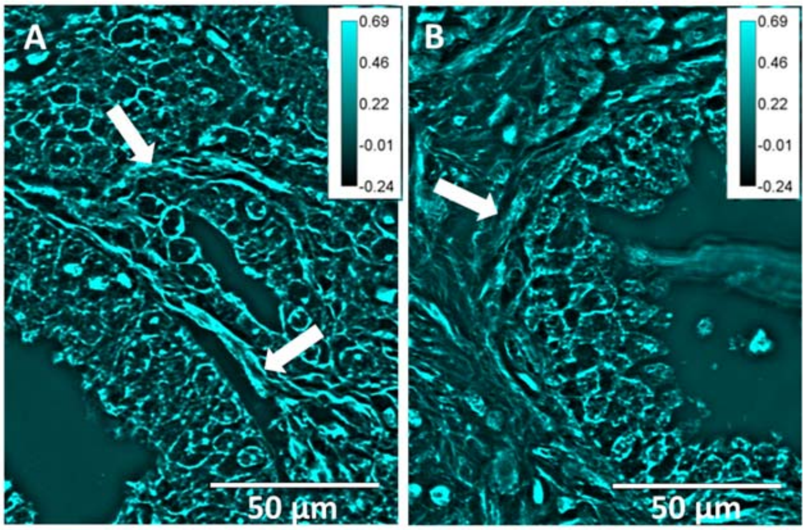

Figure S5: Effect of PSA on tissue morphology *A) Prostatectomy tissue of patient with pre-surgical PSA level 30.1 ng/ml who did not have recurrence of prostate cancer. Stromal fiber thickness is uniform but has fractionated morphology. B) Prostatectomy tissue of unmatched patient with recurrence of prostate cancer who had pre-surgical PSA level 32 ng/ml. Stromal fiber thickness is non-uniform and has a more pronounced fragmented morphology. Arrows point to such fragmented filaments.*

1. Gleason, D.F., *Classification of prostatic carcinomas.* Cancer Chemother Rep, 1966. **50**(3): p. 125-8.
2. Humphrey, P.A., *Gleason grading and prognostic factors in carcinoma of the prostate.* Mod Pathol, 2004. **17**(3): p. 292-306.
3. Epstein, J.I., et al., *Prediction of progression following radical prostatectomy. A multivariate analysis of 721 men with long-term follow-up.* Am J Surg Pathol, 1996. **20**(3): p. 286-92.
4. Partin, A.W., et al., *Combination of prostate-specific antigen, clinical stage, and Gleason score to predict pathological stage of localized prostate cancer. A multi-institutional update.* JAMA, 1997. **277**(18): p. 1445-51.
5. Egevad, L., et al., *Prognostic value of the Gleason score in prostate cancer.* BJU Int, 2002. **89**(6): p. 538-42.
6. Gleason, D.F. and G.T. Mellinger, *Prediction of prognosis for prostatic adenocarcinoma by combined histological grading and clinical staging. 1974.* J Urol, 2002. **167**(2 Pt 2): p. 953-8; discussion 959.
7. Gleason, D.F. and G.T. Mellinger, *Prediction of prognosis for prostatic adenocarcinoma by combined histological grading and clinical staging.* J Urol, 1974. **111**(1): p. 58-64.
8. D'Amico, A.V., et al., *Biochemical outcome after radical prostatectomy, external beam radiation therapy, or interstitial radiation therapy for clinically localized prostate cancer.* JAMA, 1998. **280**(11): p. 969-74.
9. Cooperberg, M.R., J.F. Hilton, and P.R. Carroll, *The CAPRA-S score: A straightforward tool for improved prediction of outcomes after radical prostatectomy.* Cancer, 2011. **117**(22): p. 5039-46.
